# Multi-Trait Phenotypic and SSR-Based Genetic Diversity Assessment of Individual Plants for Parental Selection in Turf-Type Tall Fescue

**DOI:** 10.3390/plants15203146

**Published:** 2026-10-14

**Authors:** Sinem Zere Taskın, Fikret Yonter, Sevin Teoman Duran, Meryem Ipek, Ugur Bilgili

**Affiliations:** 1 Department of Field Crops, Bursa Uludag University, 16059 Bursa, Türkiye; sinemzere@uludag.edu.tr; 2 Yıldız Plantbased Products Seeds and Agriculture Ind. Co., 59100 Istanbul, Türkiye; fikretyonter@gmail.com; 3 Organic Agriculture Program, Department of Crop and Animal Production, Karacabey Vocational School, Bursa Uludag University, 16700 Bursa, Türkiye; sevinteoman@uludag.edu.tr; 4 Department of Horticulture, Bursa Uludag University, 16059 Bursa, Türkiye; msipek@uludag.edu.tr

**Keywords:** *Schedonorus arundinaceus*, turfgrass breeding, genetic diversity, SSR markers, parental selection

## Abstract

Climate change has increased the need for turfgrass germplasm with improved adaptation, persistence, and resource-use efficiency. Tall fescue (*Schedonorus arundinaceus*) is an important cool-season turfgrass species valued for its tolerance to environmental stresses. This study aimed to assess phenotypic and molecular diversity among individual tall fescue plants derived from 24 United States Department of Agriculture (USDA) Gene Bank accessions and to identify promising turf-type parental material by integrating phenotypic and molecular information. A total of 223 genotypes were evaluated for 17 morphological and agronomic traits, and 120 genotypes were selected using a multi-trait weighted selection index for SSR-based molecular characterization. Principal component (PC) analysis showed that PC1 and PC2 accounted for 50.8% and 12.0% of the total phenotypic variation, respectively. Fourteen polymorphic SSR markers generated 69 polymorphic bands, with an overall mean polymorphism information content (PIC) value of 0.343. Unweighted Pair Group Method with Arithmetic Mean (UPGMA) analysis resolved two major molecular groups with four nested subclusters. Mantel analysis showed a weak but significant positive association between phenotypic and SSR-based molecular distances (r = 0.137, *p* < 0.001), indicating limited correspondence between the two data types. By jointly considering morphological performance and molecular diversity, 60 genotypes were identified as promising parental candidates for future tall fescue breeding programs.

## 1. Introduction

Climate change is increasingly affecting agricultural and urban ecosystems through rising temperatures, altered precipitation patterns, declining water availability, and more frequent or severe drought conditions. These impacts are also evident in Türkiye, where extensive semi-arid regions and increasing drought risk make water scarcity an important environmental concern [1]. These pressures highlight the importance of evaluating turfgrass germplasm with broad agronomic and turf-related variation for future breeding.

In this context, tall fescue is a promising cool-season turfgrass because of its relatively high drought tolerance, deep root system, persistence, and adaptability to diverse environmental conditions [2]. It is a cross-pollinated, hexaploid, perennial species widely cultivated for both forage and turf purposes [2,3], with natural populations distributed across temperate and cool regions of Europe, Northwestern Africa, North America, and Western and Central Asia [4]. Its suitability for reduced-input conditions, including lower water, fertilizer, and pesticide use, further supports its potential for sustainable turfgrass management [5].

Molecular genetic studies play a crucial role in modern plant breeding. Although classical breeding approaches are effective, they often require long selection cycles to achieve stable and improved cultivars. Molecular marker-assisted approaches can support breeding programs by enabling the identification of genetic relationships among genotypes, reducing labor requirements, and improving the efficiency of parental selection [6]. DNA-based molecular markers are particularly useful for assessing genetic diversity and relationships because they are generally independent of environmental effects [6]. Among these markers, simple sequence repeat (SSR) markers are widely used in genetic diversity studies due to their high polymorphism, codominant inheritance, reproducibility, and ease of PCR amplification [7]. In turfgrass species, SSR markers have been successfully applied in studies related to genetic diversity, association mapping, and genetic mapping [8,9,10,11].

Previous studies have used SSR markers to assess genetic diversity, population structure, and genetic relationships in tall fescue germplasm and cultivars [8,9,10,11]. Phenotypic and molecular diversity have also been evaluated together in tall fescue, and weak correspondence between these two data types has been reported [12]. Marker-based parental selection using phenotypic and SSR diversity information has also been explored in tall fescue breeding [13]. Building on these studies, the present work applies a stepwise selection strategy in turf-type germplasm, combining multi-trait phenotypic screening with SSR-based genetic diversity analysis to reduce redundancy and establish a diverse parental pool for future breeding.

Therefore, the present study aimed to assess phenotypic diversity among 223 individual tall fescue plants derived from 24 USDA Gene Bank accessions, characterize molecular diversity within the phenotypically selected subset of 120 genotypes, and identify genetically diverse and agronomically promising turf-type genotypes for use as parental material in future breeding programs.

## 2. Results

### 2.1. Morphological Characterization

The minimum, maximum, and mean values of the morphological traits recorded in the 223 evaluated tall fescue genotypes are presented in Table 1. Considerable variation was observed among the genotypes across the evaluated traits. Turf quality scores ranged from 5 to 9 across seasons, with mean values varying between 6.93 and 7.34. Similarly, seasonal color scores ranged from 5 to 9, and the highest mean color score was recorded in summer. Spring green-up scores varied from 3 to 9, indicating the presence of genotypes with different regrowth capacities after winter.

Substantial variation was also observed in seedling-stage tillering capacity, leaf texture, heading tendency, heading and flowering stages, plant diameter and autumn growth habit. Seedling-stage tillering capacity ranged from 1 to 9, while leaf texture and heading tendency also showed a wide range of variation. The 50% heading and 50% flowering stages varied between 3 and 9, indicating differences in phenological development among genotypes. Plant diameter ranged from 7 to 112 cm, while autumn growth habit scores varied from 1 to 5. Plant height ranged from 5 to 80 cm, leaf width from 0.2 to 1.4 cm, leaf length from 5.5 to 70.0 cm, panicle length from 8.0 to 35.0 cm, and last internode length from 10.0 to 54.0 cm. Regarding yield-related traits, single-plant dry biomass yield ranged from 5.0 to 475.0 g plant^−1^, whereas single-plant seed yield varied between 1.1 and 47.7 g plant^−1^ (Table S3).

In most genotypes, turf quality and seasonal color scores were above the acceptable level, indicating that a considerable proportion of the evaluated material had promising turf-type characteristics. The wide variation observed in spring green-up, tillering capacity, leaf texture, heading tendency, growth habit, plant size, biomass production, and seed yield suggests the presence of broad phenotypic diversity within the USDA-derived tall fescue material. Based on the multi-trait weighted selection index calculated from the 17 evaluated traits, 120 genotypes were selected from the total population of 223 genotypes for subsequent molecular characterization analyses (Table S5). These selected genotypes generally exhibited high turf quality and seasonal color scores, strong tillering capacity, favorable leaf texture, and desirable growth characteristics, and therefore represented the highest-ranking entries in the population.

**Figure 1:**
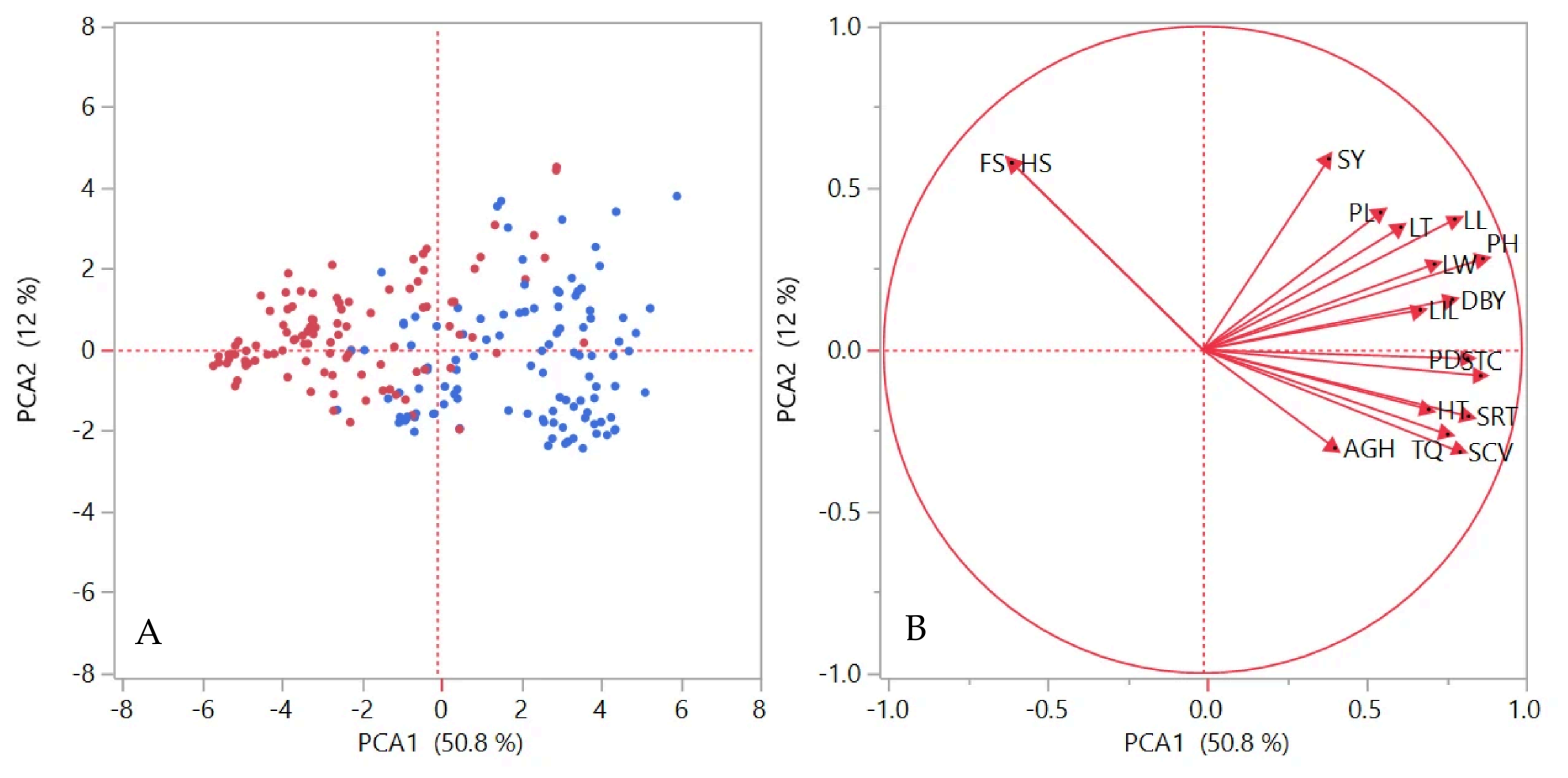
Principal component analysis (PCA) of 223 tall fescue genotypes based on 17 morphological traits. (**A**) Score plot showing the distribution of selected (blue dots; *n* = 120) and non-selected (red dots; *n* = 103) genotypes. (**B**) Loading plot illustrating the contribution and relationships of morphological traits to the first two principal components (PCA1 and PCA2). Trait abbreviations are as follows: TQ, turf quality (1–9); SCV, seasonal color variation (1–9); SRT, spring regrowth timing (1–9); STC, seedling-stage tillering capacity (1–9); LT, leaf texture (1–9); HT, heading tendency (1–9); HS, 50% heading stage (1–9); FS, 50% flowering stage (1–9); PD, plant diameter (cm); AGH, autumn growth habit (1–9); PH, plant height (cm); LW, leaf width (cm); LL, leaf length (cm); PL, panicle length (cm); LIL, last internode length (cm); DBY, single-plant dry biomass yield (g plant^−1^); and SY, single-plant seed yield (g plant^−1^).

Principal component analysis revealed that the first two principal components explained 62.8% of the total phenotypic variation among the 223 tall fescue genotypes, with PC1 and PC2 accounting for 50.8% and 12.0% of the variation, respectively. PC1 was mainly associated with turf quality, seasonal color variation, spring green-up, seedling tillering capacity, plant diameter, plant height, leaf dimensions, and dry matter yield, indicating that this component represented overall plant vigor and turf performance. In contrast, PC2 was primarily influenced by heading stage, flowering stage, seed yield, and panicle-related traits, reflecting variation in reproductive characteristics among the genotypes. The close proximity and similar orientation of heading stage (HS) and flowering stage (FS) vectors in the biplot indicated a strong positive association between these two reproductive traits. Likewise, turf quality (TQ) and seasonal color variation (SCV) were positioned in the same direction, suggesting that genotypes with superior turf quality generally exhibited better seasonal color performance (Figure 1).

### 2.2. Molecular Characterization

Molecular characterization was conducted on the 120 genotypes previously selected through the multi-trait weighted selection index; therefore, the molecular results presented below refer specifically to this phenotypically selected subset. In this study, 14 of the 24 SSR primer pairs produced clear, scorable, and polymorphic bands and were therefore used for molecular characterization (Table 2). The remaining 10 primer pairs, namely NFA073, NFA146, NFA037, NFA065, NFA021, NFA108, NFA113, NFA138, FES04, and FES13, did not produce satisfactory amplification profiles and were excluded from further analyses.

Based on the 14 polymorphic SSR primer pairs, a total of 69 polymorphic bands were detected, with an average of 4.92 bands per primer. NFA071 produced the highest number of polymorphic bands (12), whereas NFA030 produced the lowest number (2). Mean PIC values ranged from 0.210 for NFA098 to 0.416 for NFA103, with an overall mean of 0.343 (Table 2).

**Figure 2:**
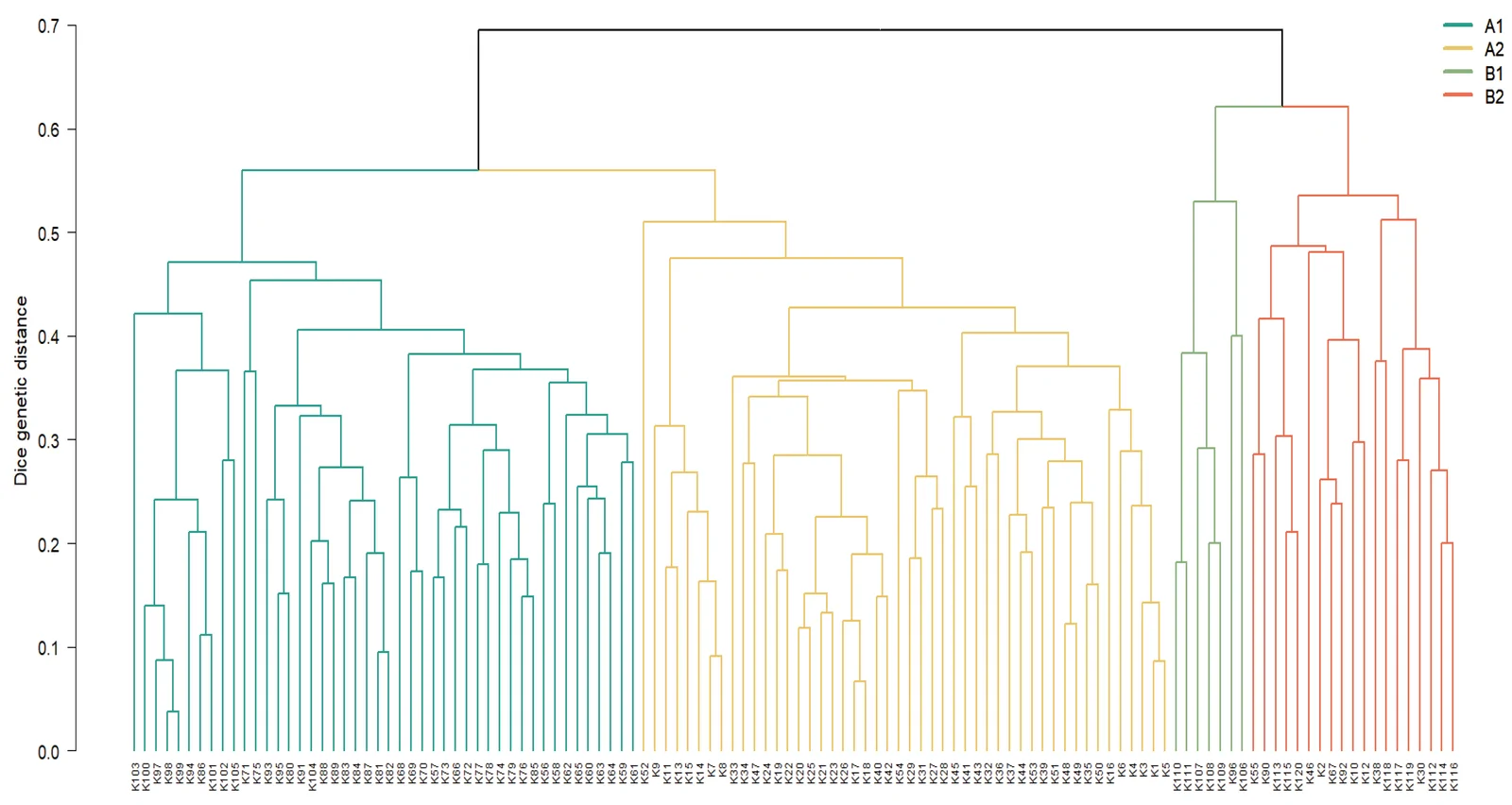
UPGMA dendrogram based on the Dice genetic distance matrix (1—Dice similarity) obtained from the molecular analysis of 120 tall fescue genotypes. A and B represent the two major molecular groups of the genotypes, while A1, A2, B1, and B2 indicate the four nested subclusters within these groups.

Among the clustering solutions evaluated from k = 2 to k = 6, the two-cluster solution showed the highest mean silhouette width (0.284) and was therefore used to define the major molecular groups. To determine genetic relationships among the 120 selected tall fescue genotypes, a genetic similarity dendrogram was constructed using the UPGMA method based on Dice genetic distances derived from the Dice similarity coefficients. The genotypes were separated into two main groups, designated as A and B, comprising 94 and 26 genotypes, respectively. Each main group was further divided into two subgroups. Group A consisted of subgroups A1 and A2 containing 46 and 48 genotypes, respectively, whereas Group B consisted of subgroups B1 and B2 containing 7 and 19 genotypes, respectively (Figure 2). This analysis resolved two major molecular groups and four nested subclusters within the evaluated germplasm. Bootstrap analysis based on 1000 marker resamplings indicated low support for the broad UPGMA divisions. Bootstrap support was 3.2% and 2.7% for the two major groups, respectively. The A1, A2, B1, and B2 subclusters showed support values of 1.7%, 7.3%, 6.2%, and 0.5%, respectively. Nevertheless, several smaller internal branches exhibited considerably higher bootstrap support, including values exceeding 70%. Thus, relationships among some closely related genotypes were reproducible, whereas the broader subdivision of the germplasm showed limited bootstrap stability.

Mantel analysis revealed a weak but significant positive association between phenotypic and SSR-based molecular distances among the 120 selected genotypes (r = 0.137, *p* < 0.001). The Spearman-based Mantel test yielded a similarly weak positive association (r = 0.123, *p* = 0.0005), supporting the robustness of the observed relationship to the choice of correlation method.

Application of the combined phenotypic–molecular selection procedure resulted in a final set of 60 parental candidates, comprising 24 genotypes from A1, 24 from A2, 3 from B1, and 9 from B2. No pair of selected genotypes showed a Dice similarity coefficient of 0.90 or higher, and the maximum pairwise similarity within the final parental set was 0.889, indicating that highly similar genotypes were effectively avoided while maintaining representation across the molecular subclusters. Accordingly, the selected genotypes were distributed as follows:

Group A1 included 24 genotypes (K79, K87, K86, K89, K83, K82, K80, K91, K99, K100, K103, K56, K61, K74, K72, K76, K78, K59, K64, K65, K62, K68, K69, K71);

Group A2 included 24 genotypes (K35, K43, K18, K44, K21, K20, K45, K22, K36, K19, K5, K54, K6, K42, K32, K37, K14, K15, K34, K16, K3, K7, K11, K9);

Group B1 included 3 genotypes (K109, K110, K106);

Group B2 included 9 genotypes (K10, K90, K67, K119, K114, K118, K115, K38, K46).

In total, 60 genotypes were selected as parental candidates for future tall fescue breeding studies.

## 3. Discussion

### 3.1. Morphological Characterization

The present study revealed substantial phenotypic variation among the evaluated individual tall fescue genotypes derived from USDA accessions, indicating considerable potential for selection within the evaluated material. Variation in turf-related and agronomic traits is particularly important for turf-type breeding, where multiple characteristics must be considered simultaneously [14,15]. Accordingly, the multi-trait weighted selection index provided a practical means of integrating these characteristics and identifying superior genotypes for subsequent molecular characterization.

PCA further demonstrated the multivariate nature of the observed phenotypic diversity. The strong contribution of dry biomass yield and plant diameter to PC1 was consistent with Pirnajmedin et al. [16], who identified dry forage yield and crown diameter as important contributors to variation in tall fescue. In addition, the close association between turf quality and seasonal color in the present study agrees with relationships among turf-related traits reported by Pirnajmedin et al. [17]. Overall, these results indicate that the evaluated germplasm contained substantial variation in both vegetative and turf-related characteristics, providing a suitable phenotypic basis for subsequent parental selection.

### 3.2. Molecular Characterization

The SSR markers used in this study revealed substantial genetic diversity among the selected tall fescue genotypes, with mean PIC values ranging from 0.210 to 0.416 and an overall mean of 0.343. These values indicate that the markers provided useful polymorphic information for distinguishing the evaluated genotypes. In total, the 14 SSR primers generated 69 polymorphic bands, ranging from 2 to 12 bands per primer. Previous studies have also demonstrated the usefulness of SSR markers for assessing genetic diversity in tall fescue and related grass species [8,9,10,18,19,20]. Differences in the levels of polymorphism reported among studies may reflect variation in the germplasm evaluated, primer sets, and marker scoring approaches. Overall, the SSR profiles obtained in the present study provided sufficient polymorphism to assess genetic relationships among the selected genotypes.

The molecular clustering of the 120 selected genotypes into two major groups and four nested subclusters indicated substantial genetic variation within the phenotypically selected subset of USDA-derived tall fescue material. High within-population variability in tall fescue has also been associated with its outcrossing nature, self-incompatibility, and hexaploid genomic constitution [10]. Population structuring among tall fescue genotypes has similarly been reported by Lou et al. [9], while Chadded et al. [21] identified distinct molecular groups using SRAP markers and related their clustering patterns primarily to geographical origin. In the present study, molecular clustering showed only partial correspondence with geographic origin and accession identity, as genotypes from the same country or accession were not always assigned to the same subcluster. Overall, these results indicate that geographic origin and accession identity alone do not fully account for the molecular relationships among the evaluated genotypes.

### 3.3. Parental Selection

The weak but significant association between phenotypic and SSR-based molecular distances indicates limited correspondence between multi-trait phenotypic variation and molecular diversity. This finding agrees with Sun et al. [12], who also reported low correspondence between morpho-physiological and SSR-based genetic distance matrices in tall fescue. The consistency of the Pearson- and Spearman-based Mantel analyses further supports this pattern. Overall, the weak correspondence between the two data types suggests that phenotypic and molecular information provide complementary perspectives for parental selection rather than interchangeable measures of diversity.

Integrating phenotypic performance with molecular information can facilitate the selection of genetically diverse parental material while reducing redundancy among breeding candidates. Molecular markers are useful for estimating genetic relationships; however, they generally reflect genome-wide variation rather than only genomic regions directly associated with target traits [22]. Therefore, phenotypic performance remains an important component of parental selection. In the present study, the integration of turf-related morphological traits, weighted selection index scores, and molecular group structure resulted in a final set of 60 genotypes combining desirable turf-type characteristics with molecular diversity. These genotypes provide a useful parental pool for future tall fescue breeding and can be further evaluated under replicated and multi-environment conditions.

## 4. Materials and Methods

### 4.1. Plant Material and Experimental Design

Tall fescue populations collected from various countries worldwide were obtained from the United States Department of Agriculture (USDA) Gene Bank, with the present study focusing on accessions originating from Türkiye and neighboring Balkan and Mediterranean countries. The plant material comprised 24 tall fescue accessions originating from seven countries, including Türkiye (7 accessions), Hungary (6), Romania (5), Greece (3), Serbia (1), Bulgaria (1), and Montenegro (1). The research commenced on 1 September 2023, and seed germination and seedling growth were monitored for a period of six months. Seeds were germinated in Petri dishes with 60 seeds per dish and subsequently transferred to plug trays. A total of 480 genotypes, representing 20 genotypes from each of the 24 accessions, were initially grown in plug trays and subsequently transplanted to the field in March 2024 at a spacing of 0.5 m × 0.5 m. Each seedling was treated as an individual seed-derived genotype. Because tall fescue is a highly outcrossing species, seedlings originating from the same accession were expected to represent genetically heterogeneous individuals rather than genetically uniform replicates. Therefore, selection in this study was conducted at the individual-plant level within accessions rather than at the accession level. Following field establishment, 223 genotypes survived and were included in the subsequent phenotypic evaluation. Accession-specific survival ranged from 0 to 100%, with an overall survival rate of 46.4% (Table S4). Survival was not considered a target trait and was not included in the multi-trait selection index. Standard cultural practices, including fertilization and irrigation, were applied throughout the experiment. In this study, a modified selection approach integrating multi-trait phenotypic evaluation and SSR-based molecular characterization was employed to identify promising parental genotypes. Mass selection is commonly preferred in cross-pollinated species because of its simplicity, ease of application, and potential to enable cultivar development within a relatively short period [23].

### 4.2. Study Area

**Figure 3:**
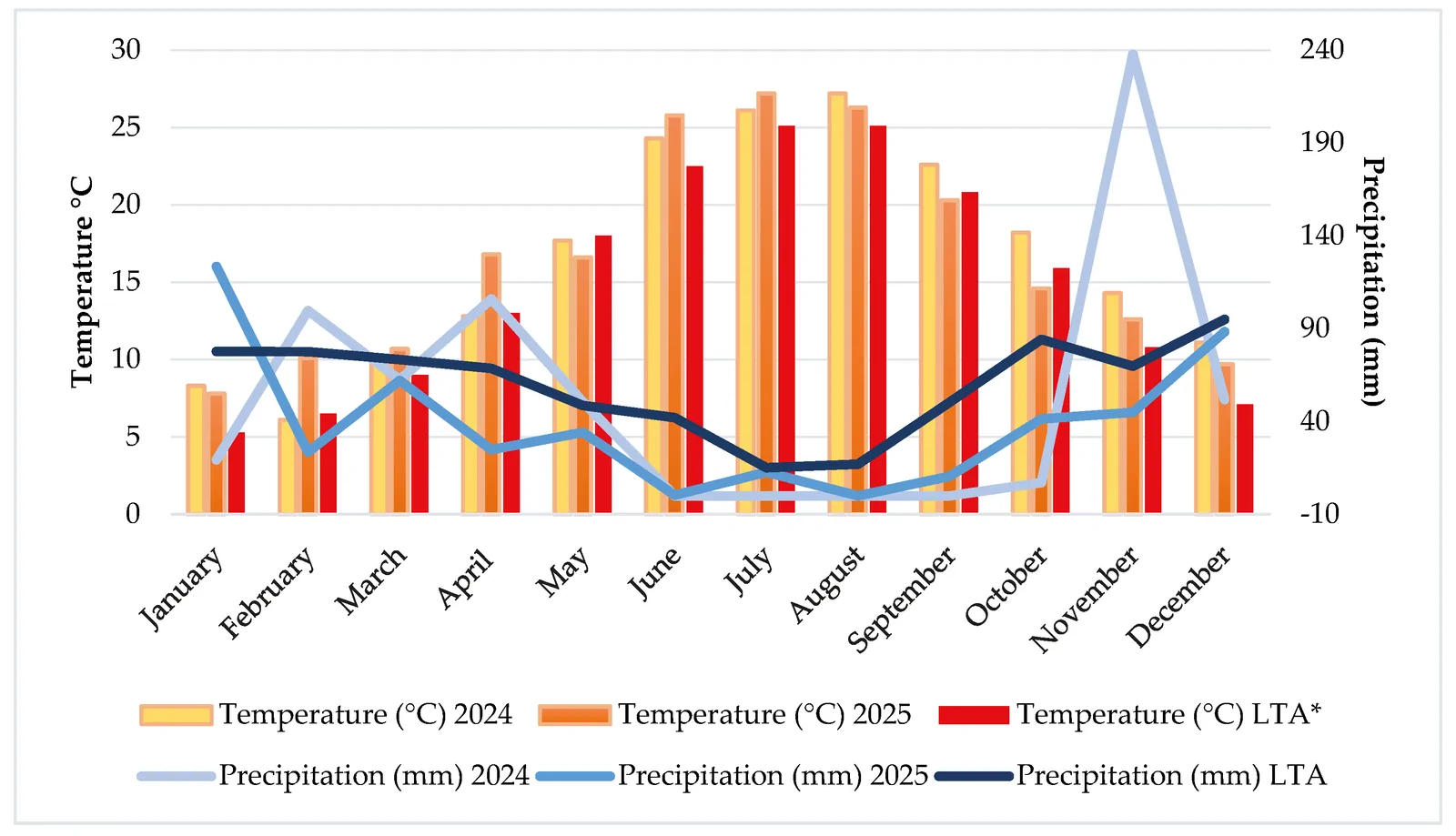
Temperature (°C) and precipitation (mm) values for Bursa Province during 2024–2025 and the long-term average. * LT: 30–year average (1990–2020).

The study was conducted at the turfgrass experimental field of the Faculty of Agriculture, Bursa Uludag University. Climate data for the experimental years and long-term averages are shown in Figure 3. During the experimental years (2024 and 2025), average air temperatures were 16.5 °C and 17.7 °C, respectively, both slightly higher than the long-term mean (14.9 °C). Total annual precipitation (634.7 mm and 280.9 mm) was lower than the long-term average (718.9 mm).

Soil samples were collected from depths of 0–20 cm and 20–40 cm at the experimental site and analyzed at the Soil Analysis Laboratory of the Department of Soil Science and Plant Nutrition, Faculty of Agriculture, Bursa Uludag University. The soil had a loam texture, consisting of 46.25% sand, 30.99% silt, and 22.76% clay, and was slightly alkaline with a pH of 8.48. Electrical conductivity was 468 µS cm^−1^, indicating non-saline soil conditions. The cation exchange capacity was 15.21 meq 100 g^−1^, lime content was 4.28%, and organic matter content was 2.09%. Total nitrogen content was low (0.106%), whereas available phosphorus (30.95 mg kg^−1^) and total potassium (5180 mg kg^−1^) levels were high.

### 4.3. Morphological Characterization

Morphological characteristics of the tall fescue genotypes were evaluated through measurements and observations of turf quality (1–9), seasonal color variation (1–9), spring green-up time (1–9), seedling-stage tillering capacity (1–9), leaf texture (1–9), heading tendency (1–9), 50% heading stage (1–9), 50% flowering stage (1–9), plant diameter (cm), autumn growth habit (1–9), plant height (cm), leaf width (cm), leaf length (cm), panicle length (cm), last internode length (cm), single-plant dry biomass yield (g plant^−1^), and single-plant seed yield (g plant^−1^).

Turf quality was visually evaluated on a 1–9 scale (1 = poorest, 6 = acceptable, 9 = superior or ideal) based on a combination of color, density, uniformity, texture, and disease or environmental stress. Turf quality was assessed on 15 July 2024 (summer), 15 October 2024 (autumn), 15 February 2025 (winter), and 15 April 2025 (spring). Seasonal color was evaluated on the same dates using a 1–9 scale, with 1 representing straw-yellow/brown and 9 representing dark green. Seasonal color was used to distinguish color differences associated with disease or insect damage, nutrient deficiencies, and environmental stress [24]. Spring green-up was visually evaluated in April 2025 on a 1–9 scale based on the degree of green growth observed in spring, where 1 represented plants that had not greened up or were dead, 5 represented intermediate green-up, and 9 represented complete and vigorous green-up. Seedling-stage tillering capacity was evaluated in spring 2024 and rated from 1 (very low) to 9 (very high). Leaf texture was visually evaluated on 10 July 2024 using a 1–9 scale (1 = narrow/fine, 5 = medium, 9 = wide/coarse) based on the last leaf of a representative culm. On the same date, plant diameter, plant height, leaf width, leaf length, and last internode length were also determined. Plant diameter (cm) was calculated as the average of horizontal and vertical canopy spread. Plant height (cm) was measured from the soil surface to the highest point of the plant, while leaf width (cm) and leaf length (cm) of the uppermost leaf were recorded. The last internode length (cm) was determined by measuring the distance between the last internode and the uppermost spikelet. Heading tendency, 50% heading stage, 50% flowering stage, and panicle length were evaluated during April–May 2024. Heading tendency was recorded when at least three panicles were present and scored from 1 (very weak) to 9 (very strong). The dates on which 50% of each individual plant reached heading and flowering were recorded and subsequently converted to a 1–9 ordinal earliness–lateness scale based on the relative phenological timing among genotypes, with lower scores representing earlier development and higher scores representing later development (1 = very early, 5 = intermediate, and 9 = late). Panicle length (cm) was measured between the lowest and uppermost spikelets. Autumn growth habit was assessed on 10 October 2024 using a 1–9 scale (1 = erect, 3 = semi-erect, 5 = intermediate, 7 = semi-prostrate, 9 = prostrate). Single-plant dry biomass yield was determined on 30 May 2024. Plants were harvested individually at the heading stage, dried at 105 °C for 48 h, and weighed to determine dry biomass yield. After cutting, the same plants were allowed to regrow and develop reproductive structures, and single-plant seed yield was subsequently determined in spring 2025 on a gram basis (Figure S1) [24,25,26].

### 4.4. Molecular Characterization

DNA was isolated following the modified cetyltrimethylammonium bromide (CTAB) method described by Futterer et al. [27]. Fresh leaf samples were collected from the selected 120 tall fescue genotypes (Table S5). The leaf samples were transported to the laboratory and placed in a Christ Alpha 1–4 LD Plus lyophilizer (Martin Christ Gefriertrocknungsanlagen GmbH., Ronkonkoma, NY, USA), where they were dried for three days. The dried leaf samples were ground into a fine powder and subsequently used for DNA isolation. After DNA isolation, DNA concentrations were determined using a Qubit fluorometer (Invitrogen, Carlsbad, CA, USA), and the samples were diluted with TE buffer to a final concentration of 50 ng µL^−1^ (Table S1). DNA samples were subsequently visualized on 0.7% agarose gel.

In this study, 24 SSR primer pairs developed by Saha et al. [19] and Ustariz et al. [28] were used (Table S2). SSR analyses were performed using the three-primer method described by Schuelke [29]. According to this method, sequence-specific reverse primers for the target regions, M13 primers labeled with LI-COR infrared dyes at 700 or 800 nm (LI-COR, Lincoln, NE, USA), and forward primers containing the M13 tail sequence (5′-GACGTTGTAAAACGACGGCC) were employed. Amplification reactions were performed in a final volume of 20.35 µL, following the three-primer PCR method described by Schuelke [29]. Each PCR mixture contained 2.0 µL of genomic DNA (100 ng), 2.0 µL of 10× PCR buffer, 2.0 µL of dNTPs (0.25 mM of each at final concentration), 1.6 µL of MgCl_2_ (2 mM), 1.0 µL of the forward primer (0.10 µM), 1.0 µL of the reverse primer (0.20 µM), 0.8 µL of the M13 primer (0.20 µM), 0.5 µL of Taq DNA polymerase (0.1 U), and 9.45 µL of nuclease-free water. PCR amplifications were carried out with an initial denaturation step at 95 °C for 3 min, followed by 5 cycles of 30 s at 95 °C, 1 min at 62 °C (annealing temperature was gradually decreased 1 °C at each cycle), and 1 min at 72 °C. This was followed by 28 cycles consisting of 30 s at 95 °C, 1 min at 57 °C, and 1 min at 72 °C, and subsequently 7 additional cycles of 30 s at 95 °C, 1 min at 54 °C, and 1 min at 72 °C, with a final extension step at 72 °C for 5 min. Annealing temperatures of primers ranged between 57 and 62 °C depending on the melting temperatures of the primers. PCR products were separated on 6% polyacrylamide gels using a LI-COR 4200 DNA analyzer (Lincoln, NE, USA) at 30 W and 35 °C for 2.5 h, and SSR fragments were visualized using SAGA-GT™ v3.3.0 software (LI-COR, Lincoln, NE, USA).

### 4.5. Statistical Analysis

A multi-trait weighted selection index was calculated using the 17 evaluated morphological and agronomic traits. Each trait was first normalized to a 0–1 scale using min–max standardization. For traits in which higher values were considered desirable, normalized scores were calculated as:

$$S_{i}=\frac{X_{i}-X_{\min}}{X_{\max}-X_{\min}}$$

For traits in which lower values were considered desirable, normalized scores were calculated as:

$$S_{i}=\frac{X_{\max}-X_{i}}{X_{\max}-X_{\min}}$$

where *X_i_* is the observed value of a genotype for a given trait, and *X_min_* and *X_max_* represent the minimum and maximum values observed for that trait across all genotypes.

The overall selection index was calculated as:

$$\mathrm{Selection}\;\mathrm{Index}=\sum_{i=1}^{n}\left(w_{i}\times S_{i}\right)\times 100$$

where *w_i_* is the weight assigned to trait *i*, *S_i_* is the normalized score of trait *i*, and *n* is the total number of evaluated traits. Trait weights were determined according to their relative importance in turfgrass breeding, with greater emphasis placed on turf quality, seasonal color variation, spring regrowth timing, seedling-stage tillering capacity, single-plant dry biomass yield, and single-plant seed yield. Higher weights were assigned to turf quality and seasonal color because these traits directly represent the primary aesthetic and functional objectives of turf-type breeding. Spring regrowth and tillering capacity were also emphasized because rapid post-winter recovery and dense sward formation are desirable, while lower biomass production was favored to reduce mowing requirements and seed yield was retained as an indicator of reproductive potential (Table 3). Genotypes were ranked according to their final index scores, and the highest-ranking 120 genotypes were selected for subsequent molecular characterization analyses. This set represented a phenotypically selected subset rather than a random sample of the 223 genotypes, and SSR analysis was therefore used to assess molecular diversity and reduce genetic redundancy within this selected group. The index was conceptually based on multi-trait selection approaches commonly used in plant breeding, in which multiple traits are integrated into a single selection criterion to facilitate genotype ranking and selection [30].

Principal component analysis (PCA) was performed using the 17 morphological and agronomic traits of the 223 tall fescue genotypes. Seasonal mean values were used for turf quality and seasonal color variation. PCA was conducted based on the correlation matrix using standardized variables, and the first two principal components were used to generate score and loading plots showing genotype distribution and trait relationships. Analyses were performed using JMP Pro 13 (SAS Institute Inc., Cary, NC, USA).

Following visualization of the gel images, SSR fragments were scored in binary form, with 1 indicating the presence and 0 indicating the absence of a reproducible band. Because tall fescue is a hexaploid species and allele dosage could not be reliably determined from the banding profiles, each polymorphic band was treated as a binary marker, following the approach used for SSR analysis in tall fescue by Fu et al. [10]. For each band, the frequency of band presence (*p*) was calculated across genotypes, and polymorphism information content (PIC) was calculated as 2*p*(1 − *p*) according to Roldán-Ruiz et al. [31]. Primer-level PIC values were obtained as the mean PIC values of the polymorphic bands generated by each primer.

Pairwise genetic similarity among the 120 tall fescue genotypes was calculated using the Dice similarity coefficient [32], and the corresponding genetic distance matrix was obtained as 1—Dice similarity.

Genetic relationships among the genotypes were visualized by a UPGMA (Unweighted Pair Group Method with Arithmetic Mean) dendrogram constructed from the Dice genetic distance matrix in R. To determine the major clustering pattern, solutions ranging from k = 2 to k = 6 were evaluated using mean silhouette width with the cluster package, and the solution with the highest mean silhouette width was selected. The resulting major clusters were designated as A and B. To characterize finer hierarchical structure within these major clusters, the dendrogram was additionally partitioned into four nested subclusters, designated A1, A2, B1, and B2.

The robustness of the UPGMA dendrogram was evaluated by bootstrap analysis with 1000 resamplings of the binary marker bands. For each bootstrap replicate, the Dice genetic distance matrix was recalculated and a UPGMA tree was reconstructed. Bootstrap support values were then calculated for the corresponding branches of the original dendrogram. To assess the relationship between phenotypic and molecular variation, a Mantel test was performed between the Euclidean distance matrix calculated from the standardized 17 phenotypic traits and the SSR-based Dice genetic distance matrix. The analysis was conducted in R using the vegan package, employing Pearson correlation as the primary correlation method. Statistical significance was evaluated using 9999 permutations. A Spearman-based Mantel test was additionally performed to assess the robustness of the observed association to the choice of correlation method.

A final parental set of 60 genotypes, corresponding to 50% of the 120 SSR-characterized genotypes, was defined to obtain a manageable breeding population while retaining broad phenotypic and molecular diversity. The final parental set was selected to retain representation from all four molecular subclusters and comprised 24 genotypes from A1, 24 from A2, 3 from B1, and 9 from B2. Within each subcluster, genotypes with higher multi-trait weighted selection index scores were prioritized. To minimize molecular redundancy, a pairwise Dice similarity coefficient of ≥0.90 was adopted as an operational threshold for high genetic similarity; when highly similar candidates occurred, a single representative was retained by jointly considering weighted index performance and subcluster representation.

## 5. Conclusions

In this study, 223 individual tall fescue plants derived from 24 USDA Gene Bank accessions were evaluated using 17 morphological and agronomic traits, and a phenotypically selected subset of 120 genotypes was subsequently subjected to SSR analysis. The phenotypic and molecular analyses showed clear variation among the genotypes. The weak but significant relationship between phenotypic and molecular distances showed that both types of data are useful for parental selection. By considering phenotypic performance and molecular diversity together, 60 genotypes were selected as promising parental candidates. These genotypes may provide useful parental material for future tall fescue breeding programs and could be further evaluated under replicated, multi-environment, and stress-related conditions.

## Figures and Tables

**Table 1:** Minimum, maximum, and mean values of morphological traits evaluated in 223 tall fescue genotypes.

No	Morphologic Traits	Minimum	Maximum	Mean ± SD
1 *	Turf quality (winter)	5.0	9.0	6.93 ± 1.24
	Turf quality (spring)	5.0	9.0	6.96 ± 1.00
	Turf quality (summer)	5.0	9.0	7.34 ± 1.28
	Turf quality (autumn)	5.0	9.0	6.93 ± 1.24
2	Seasonal color (winter)	5.0	9.0	7.16 ± 1.24
	Seasonal color (spring)	5.0	9.0	7.23 ± 0.82
	Seasonal color (summer)	5.0	9.0	7.43 ± 1.20
	Seasonal color (autumn)	5.0	9.0	7.15 ± 1.25
3	Spring green-up	3.0	9.0	7.57 ± 1.42
4	Seedling-stage tillering	1.0	9.0	7.48 ± 1.44
5	Leaf texture	1.0	9.0	6.47 ± 1.35
6	Heading tendency	1.0	9.0	5.98 ± 2.89
7	50% heading stage	3.0	9.0	6.71 ± 2.48
8	50% flowering stage	3.0	9.0	6.71 ± 2.48
9	Plant diameter (cm)	7.0	112.0	44.34 ± 23.10
10	Autumn growth habit	1.0	5.0	3.18 ± 0.71
11	Plant height (cm)	5.0	80.0	33.78 ± 16.64
12	Leaf width (cm)	0.2	1.4	0.79 ± 0.22
13	Leaf length (cm)	5.5	70.0	28.26 ± 10.92
14	Panicle length (cm)	8.0	35.0	19.37 ± 5.31
15	Last internode length (cm)	10.0	54.0	25.58 ± 9.43
16	Single-plant dry biomass yield (g)	5.0	475.0	107.87 ± 100.79
17	Single-plant seed yield (g)	1.1	47.7	6.21 ± 5.57

* 1. Turf quality (1–9), 2. Seasonal color variation (1–9), 3. Spring regrowth timing (1–9) 4. Seedling-stage tillering capacity (1–9), 5. Leaf texture (1–9), 6. Heading tendency (1–9), 7. 50% heading stage (1–9), 8. 50% flowering stage (1–9), 9. Plant diameter (cm), 10. Autumn growth habit (1–9), 11. Plant height (cm), 12. Leaf width (cm), 13. Leaf length (cm), 14. Panicle length (cm), 15. Last internode length (cm), 16. Single-plant dry biomass yield (g plant^−1^), 17. Single-plant seed yield (g plant^−1^).

**Table 2:** Number of polymorphic bands and mean polymorphism information content (PIC) values of the SSR primers.

SSR Markers	Polymorphic Bands	PIC
NFA150	6	0.219
NFA133	5	0.393
NFA012	6	0.368
NFA071	12	0.388
NFA103	5	0.416
NFA140	6	0.398
NFA142	4	0.340
NFA030	2	0.392
NFA098	3	0.210
NFA078	3	0.404
NFA048	4	0.272
NFA046	4	0.400
NFA120	6	0.250
FES24	3	0.304
Total/Mean	69	0.343

**Table 3:** Trait weights used in the multi-trait weighted selection index.

No	Trait	Direction	Weight
1	Turf quality	Higher is better	0.250
2	Seasonal color variation	Higher is better	0.250
3	Spring regrowth timing	Higher is better	0.100
4	Seedling-stage tillering capacity	Higher is better	0.100
5	Leaf texture	Lower is better	0.050
6	Heading tendency	Higher is better	0.010
7	50% heading stage	Higher is better	0.010
8	50% flowering stage	Higher is better	0.010
9	Plant diameter	Higher is better	0.010
10	Autumn growth habit	Higher is better	0.010
11	Plant height (cm)	Lower is better	0.010
12	Leaf width (cm)	Lower is better	0.010
13	Leaf length (cm)	Lower is better	0.010
14	Panicle length (cm)	Higher is better	0.010
15	Last internode length (cm)	Higher is better	0.010
16	Single-plant dry biomass yield	Lower is better	0.100
17	Single-plant seed yield	Higher is better	0.050
		Total weight	1.000

## Data Availability

All data generated or analyzed during this study are included in this article and its Supplementary Materials.

## Supplementary Materials

The following supporting information can be downloaded at https://www.mdpi.com/article/10.3390/plants15203146/s1: Table S1: DNA concentration of tall fescue genotypes used for molecular analysis; Table S2: Primers and their sequences used in the study; Table S3: Results of measurements and observations from the morphological characterization of 223 tall fescue genotypes; Table S4: USDA accession numbers and countries of origin of the 120 tall fescue genotypes selected for SSR analysis; Table S5: Ranking of 223 tall fescue genotypes based on the multi-trait weighted selection index; Figure S1: Representative images and schematic illustrations showing the evaluation methods for the 17 phenotypic and turf-related traits assessed in tall fescue.
