# Supplementary material for: Multi-Trait Phenotypic and SSR-Based Genetic Diversity Assessment of Individual Plants for Parental Selection in Turf-Type Tall Fescue
Source: Plants (Basel). 2026 Oct 14;15(20):3146. doi: 10.3390/plants15203146 (PMC13708742; doi:10.3390/plants15203146)
Supplement: Supplementary file 1 [file plants-15-03146-s001.zip › plants-4620721-supplementary.pdf]

Table S1. DNA concentration of tall fescue genotypes used for molecular analysis.

Genotype code	DNA Concentration (ng/μL)	Genotype code	DNA Concentration (ng/μL)	Genotype code	DNA Concentration (ng/μL)
**K1**	452	**K41**	550	**K81**	540
**K2**	660	**K42**	650	**K82**	434
**K3**	800	**K43**	465	**K83**	441
**K4**	880	**K44**	451	**K84**	750
**K5**	750	**K45**	440	**K85**	369
**K6**	570	**K46**	800	**K86**	366
**K7**	560	**K47**	870	**K87**	570
**K8**	477	**K48**	570	**K88**	303
**K9**	800	**K49**	497	**K89**	690
**K10**	730	**K50**	630	**K90**	380
**K11**	780	**K51**	477	**K91**	307
**K12**	620	**K52**	290	**K92**	250
**K13**	820	**K53**	156	**K93**	412
**K14**	510	**K54**	350	**K94**	348
**K15**	570	**K55**	303	**K95**	313
**K16**	447	**K56**	530	**K96**	335
**K17**	496	**K57**	510	**K97**	205
**K18**	530	**K58**	495	**K98**	360
**K19**	281	**K59**	630	**K99**	349
**K20**	960	**K60**	550	**K100**	429
**K21**	450	**K61**	436	**K101**	119
**K22**	302	**K62**	660	**K102**	365
**K23**	434	**K63**	305	**K103**	216
**K24**	359	**K64**	540	**K104**	225
**K25**	293	**K65**	362	**K105**	223
**K26**	474	**K66**	285	**K106**	291
**K27**	540	**K67**	492	**K107**	159
**K28**	280	**K68**	840	**K108**	266
**K29**	424	**K69**	457	**K109**	195
**K30**	450	**K70**	730	**K110**	176
**K31**	456	**K71**	830	**K111**	165
**K32**	790	**K72**	650	**K112**	247
**K33**	140	**K73**	438	**K113**	274
**K34**	660	**K74**	498	**K114**	455
**K35**	1000	**K75**	486	**K115**	226
**K36**	363	**K76**	800	**K116**	145
**K37**	960	**K77**	380	**K117**	406
**K38**	41	**K78**	448	**K118**	620
**K39**	790	**K79**	570	**K119**	238
**K40**	404	**K80**	182	**K120**	279

Table S2. Primers and their sequences used in the study.

No	Primer name	Forward primer sequence (F)	Reverse primer sequence (R)	References
**1**	**NFA037**	GCATCGACCTACCCTGACTA	GTGTCCCTGGCGATCTTG	(Saha et al., 2004)
**2**	**NFA065**	GGATGGATCCTCACAATGCT	CTCCTCCTCTCCTCCAGCTC	
**3**	**NFA146**	GGAAGCAAAATGATAGGATTGG	ATAGCGCATGACTGTTGATGAT	
**4**	**NFA113**	CTCGAAGTAGGCGGAGATGAC	AGTACGAGGAGGGGATGGAGAT	
**5**	**NFA021**	CACAGCTCGTATAGGCGTCA	CTTGTCGAAGAGCGGGAAC	
**6**	**NFA150**	TGCAGTCGGTTAAGATCAAGAA	GCAGAGCAATGGAGAGGTC	
**7**	**NFA133**	GCATCTAGGGTTTCCTCCCGAGT	TGACTGGCTTGAGCTGATCTAC	
**8**	**NFA012**	TTGCCAGGAAATTTTGCTCT	ATGGTATTGGCAGCAAGAGG	
**9**	**NFA071**	TCCTAAGCAGAGCTCGATCC	GAGGTTGGCGAACTTCCTC	
**10**	**NFA103**	GCAAGACATCACATAGCTGTCG	CTCGTACCGCCCCACAAG	
**11**	**NFA108**	ATCAAACCCTGAGAAGCAAAAC	GTTCCGTCGCGTACATTCTT	
**12**	**NFA138**	GCCTTGGAGAAGAATATCCACA	CAGTGGGGAATTGCAGATAGAT	
**13**	**NFA140**	CTCACAAAGCATCAACCAAGTC	GGAGTACCAGCAGTACCAGGAC	
**14**	**NFA142**	CTTTGGACAAGGCAATGGAAT	GTTGTTCTTCTGCGGGTAGTC	
**15**	**NFA030**	AGTCGGTGGTGAAGCTGAAG	ACAACTAGGGGGCTGGTCA	
**16**	**NFA098**	TCTGCAAGGTCACTGGATCA	GGAGCAAGAAGGACGGAGAC	
**17**	**NFA078**	GTTCTTGGCGTGCTTCTTCTT	CGGGAGCTACAACAATAGCAG	
**18**	**NFA073**	TTCCTTCCTCTTTCCCGAAC	ATGGTCTCCCTCTGCTCGTA	
**19**	**NFA048**	CAGGCTGTTAACGGTGTCCT	CCTTCTTCTTGGGAGGGAAA	
**20**	**NFA046**	GCATCGACCTACCCTGACTA	GTGTCCCTGGCGATCTTG	
**21**	**NFA120**	TGTTGGTAGTATTCGCTTTTGG	GAGGATTCGGTCCCTTGTATC	
**22**	**FES13**	GCAATCTTTCCTTGCATCATCT	AGCAGCCTCAACAACTCCAG	(Ustariz et al., 2022)
**23**	**FES04**	GTTGAGTGGAGTTGAGGTCACA	AAAGGAACGAGGAAACACTACG	
**24**	**FES24**	CTTACACCAGGAAACGGAAGAT	TGGAGAAGCAAAATGTGAAGTG	

Table S3. Results of measurements and observations from the morphological characterization of 223 tall fescue genotypes.

Genotype codes	1. Turf quality				2. Seasonal color variation				3. Spring regrowth timing	4. Seedling-stage tillering capacity	5. Leaf texture	6. Heading tendency	7. 50% heading stage	8. 50% flowering stage	9. Plant diameter	10. Autumn growth habit	11. Plant height (cm)	12. Leaf width (cm)	13. Leaf length (cm)	14. Panicle length (cm)	15. Last internode length (cm)	16. Single-plant dry biomass yield (g plant^{-1})	17. Single-plant seed yield (g plant^{-1})
	winter	spring	summer	autumn	winter	spring	summer	autumn															
G1	6	6	5	6	7	7	7	7	6	6	7	7	5	5	22	3	20	0.6	17	17	29	25	7.0
K1	7	8	8	7	8	7	8	8	6	7	7	7	5	5	27	3	40	0.8	35	27	41	90	11.8
K2	8	8	7	8	8	7	9	8	6	7	7	7	5	5	30	3	45	1.0	32	22	40	90	8.3
K3	8	7	8	8	8	7	9	8	6	6	7	7	5	5	38	3	35	0.8	25	22	40	55	16.5
K4	7	7	9	7	7	7	9	7	8	7	5	7	5	5	31	3	25	0.6	25	18	24	85	3.2
K5	7	8	9	7	8	8	9	8	7	7	7	8	5	5	42	3	40	0.8	31	26	28	160	13.5
G7	5	7	7	5	5	8	7	5	7	7	9	1	9	9	85	3	40	0.8	35	22	27	45	3.9
K6	7	8	9	7	8	8	9	8	7	7	7	8	5	5	26	3	45	0.9	38	27	19	175	16.1
K7	8	8	8	8	9	8	8	9	9	9	7	9	9	9	67	5	79	1.0	36	23	35	210	3.4
K8	7	8	8	7	8	8	8	8	7	7	8	8	5	5	37	3	42	1.1	32	22	32	185	24.2
K9	8	7	9	8	9	7	9	9	9	9	7	9	3	3	84	5	44	0.8	31	18	37	175	3.4
K10	8	8	9	8	7	8	9	7	9	8	6	7	9	9	43	3	30	0.6	30	17	14	85	3.4
K11	8	8	9	8	7	8	9	7	9	8	6	7	9	9	44	3	30	0.6	30	17	14	85	3.4
G14	6	6	7	6	6	7	7	6	6	6	9	9	9	9	58	3	27	1.0	36	17	35	14	12.5
K12	8	8	9	8	7	8	9	7	9	8	6	7	9	9	41	3	30	0.6	30	17	14	85	3.4
K13	8	8	9	8	7	8	9	7	9	8	6	9	5	5	43	3	30	0.7	28	15	14	95	3.5
K14	8	8	9	8	7	8	9	7	9	8	6	9	5	5	43	3	30	1	28	15	14	95	3.2
G18	5	7	7	5	5	7	7	5	3	1	1	2	9	9	42	3	40	0.9	46	17	19	46	3.4
K15	8	8	9	8	8	8	9	8	8	8	8	8	5	5	112	3	40	1.0	32	24	39	195	20.2
K16	8	9	9	8	8	7	6	8	9	9	9	9	5	5	57	3	70	1.1	70	35	30	415	24.9
K17	6	9	8	6	6	9	8	6	8	9	9	2	9	9	60	3	50	1.1	40	32	31	75	8.8
K18	8	8	9	8	7	8	9	7	9	8	7	2	5	5	39	3	31	0.7	23	15	14	65	3.4
G23	6	9	8	6	6	9	7	6	8	9	9	2	9	9	63	3	60	1.2	54	32	39	155	17.2
K19	8	8	9	8	7	8	9	7	9	8	7	2	5	5	41	3	31	0.7	23	15	14	65	3.5
G25	6	8	6	6	6	9	7	6	8	9	9	1	9	9	26	3	35	0.7	28	22	28	35	4.3
K20	8	8	9	8	7	8	9	7	9	8	7	2	5	5	39	3	31	0.7	23	15	14	65	3.4
K21	6	8	9	6	6	8	8	6	8	9	9	1	9	9	37	3	70	1.2	49	18	19	90	18.4
K22	8	8	9	8	7	8	9	7	8	8	6	1	5	5	35	3	30	0.6	21	15	14	45	4.6
K23	6	8	8	6	6	8	8	6	8	9	9	3	9	9	37	3	50	0.9	48	25	40	60	20.3
K24	8	8	8	8	8	8	8	8	7	8	6	8	5	5	35	3	28	0.6	27	24	28	105	3.4
K25	9	6	7	9	9	7	7	9	9	9	7	9	3	3	90	5	40	1	36	21	36	315	3.3
K26	8	5	7	8	9	5	8	9	9	9	7	9	3	3	60	3	46	0.8	27	17	47	55	3.6
K27	7	7	9	7	7	7	9	7	8	7	5	5	5	5	36	3	24	0.7	19	18	25	60	3.8
G34	6	6	7	6	7	7	7	7	7	8	6	5	5	5	49	3	35	0.7	44	27	39	75	3.4
K28	7	7	9	7	7	7	9	7	8	7	5	5	5	5	36	3	24	0.7	19	18	25	60	3.2

K29	7	7	9	7	7	7	9	7	8	7	5	5	5	5	22	3	24	0.7	19	18	25	60	3.5
K30	6	8	8	6	7	8	8	7	9	8	8	9	9	9	90	3	50	1.0	38	17	23	150	19.5
K31	6	8	8	6	7	8	8	7	9	9	8	9	5	5	43	3	60	0.9	46	29	38	130	17.1
K32	7	7	7	7	7	7	8	7	9	9	6	9	9	9	61	3	35	1.0	21	17	21	34	18.3
K33	9	5	6	9	9	5	6	9	9	9	6	9	3	3	74	3	40	0.8	47	27	33	46	7.2
K34	9	7	8	9	9	7	8	9	9	9	7	9	3	3	51	3	65	1.0	52	24	27	335	11.6
K35	9	7	7	9	9	7	8	9	9	9	7	9	9	9	59	3	50	1.2	36	21	25	78	6.5
K36	9	6	8	9	9	6	7	9	9	9	7	9	3	3	105	3	70	1.0	43	24	46	178	14.9
K37	8	7	8	8	9	7	8	9	9	8	8	9	3	3	69	3	50	1.1	38	22	29	315	3.4
K38	8	7	8	8	9	7	8	9	9	9	8	9	3	3	86	3	45	1.3	36	23	16	165	13.2
K39	7	9	9	7	7	9	9	7	9	9	9	9	9	9	85	3	70	1.0	38	16	19	465	5.7
K40	7	9	9	7	7	9	9	7	9	9	9	9	9	9	75	3	80	1.2	54	24	38	140	17.9
K41	8	6	8	8	9	6	9	9	9	9	9	9	9	9	72	3	60	1.2	48	21	30	350	11.2
K42	8	9	8	8	9	9	8	9	9	9	9	9	9	9	67	3	67	1.1	45	22	24	350	9.7
K43	8	7	9	8	9	8	8	9	9	9	7	9	3	3	73	3	58	1.1	46	27	34	155	9.0
K44	8	7	9	8	9	8	8	9	9	9	7	9	9	9	93	3	57	1.0	42	24	27	290	6.6
K45	8	7	9	8	9	8	8	9	9	9	7	9	3	3	70	3	70	0.9	46	29	34	310	7.1
K46	8	7	7	8	9	7	7	9	9	9	7	9	9	9	80	3	40	0.9	40	26	28	105	3.3
K47	8	5	9	8	9	6	7	9	9	9	7	9	3	3	86	3	65	1.4	46	20	29	475	17.5
K48	8	5	9	8	9	6	7	9	9	9	7	9	3	3	72	3	55	0.9	53	24	40	355	16.5
K49	8	5	7	8	8	7	7	8	9	9	5	9	3	3	61	3	60	1.2	47	23	33	245	8.0
K50	8	7	7	8	8	7	8	8	9	9	5	9	9	9	81	3	60	0.9	47	24	31	185	3.6
K51	7	7	9	7	7	7	9	7	8	7	5	7	5	5	31	3	25	0.6	25	18	24	85	4.7
K52	7	7	9	7	7	7	9	7	8	7	5	7	5	5	31	3	25	0.6	25	18	24	85	3.4
K53	7	9	8	7	7	8	8	7	9	9	7	9	9	9	42	3	70	0.9	49	23	29	270	24.5
K54	6	7	6	6	6	7	7	5	7	7	8	1	9	9	38	3	37	0.9	27	22	28	30	4.6
K55	7	8	7	7	8	8	8	8	6	5	5	5	9	9	66	3	30	0.6	16	16	27	27	9.0
K56	9	8	8	9	9	8	8	9	9	9	7	9	9	9	66	3	63	1.0	50	22	25	310	6.8
K57	8	6	7	8	9	7	7	9	9	9	7	9	3	3	68	5	40	0.9	30	23	46	260	5.1
K58	8	6	6	8	9	7	7	9	9	9	7	9	3	3	65	5	48	0.9	29	24	27	110	3.8
K59	8	6	8	8	9	7	9	9	9	9	7	9	3	3	90	5	37	1.0	30	20	24	90	4.2
K60	8	6	8	8	9	7	8	9	9	9	7	9	9	9	79	3	49	0.9	35	23	34	345	10.0
K61	8	6	8	8	9	7	8	9	9	9	7	9	3	3	92	5	45	0.9	32	17	44	240	3.4
K62	8	6	8	8	9	7	8	9	9	9	7	9	9	9	84	3	55	0.9	43	27	35	215	4.4
K63	8	7	8	8	9	7	8	9	9	9	8	9	3	3	84	3	47	1.0	30	21	40	265	5.3
K64	8	8	8	8	9	8	8	9	9	9	8	9	9	9	70	5	47	0.8	24	21	32	115	2.9
K65	8	8	9	8	9	8	9	9	9	9	9	9	3	3	72	5	50	1.0	31	18	39	145	6.7
G73	6	6	7	6	6	7	7	6	6	6	7	6	5	5	54	3	27	0.9	25	17	19	50	9.1
K66	8	7	9	8	9	7	9	9	9	9	8	9	3	3	73	3	44	1.0	37	15	28	195	5.1
G75	6	7	8	6	6	7	8	6	7	7	8	8	5	5	32	3	37	0.8	27	22	28	95	11.7
K67	9	7	7	9	9	7	7	9	9	9	7	9	9	9	82	5	42	0.9	32	24	35	190	4.1
K68	9	7	9	9	9	7	9	9	9	9	7	9	3	3	80	5	50	1.0	32	21	36	275	3.8
K69	9	8	9	9	9	8	9	9	9	9	7	9	9	9	71	5	50	1.0	36	21	40	165	4.1
K70	9	8	9	9	9	8	9	9	9	9	7	9	3	3	65	5	40	1.2	34	22	44	160	3.4
K71	9	6	8	9	9	7	8	9	9	9	7	9	3	3	59	5	37	1.0	34	16	36	140	4.3
G81	6	9	8	6	6	9	7	6	9	9	9	1	9	9	86	3	70	1	40	20	34	245	6

K72	8	7	7	8	9	7	8	9	9	9	8	8	3	3	53	5	35	1.0	31	18	43	100	4.0
K73	9	7	7	9	9	7	8	9	9	9	8	9	3	3	84	5	48	0.9	29	28	54	380	4.7
K74	9	6	8	9	9	7	8	9	9	9	8	8	3	3	74	5	36	1.1	27	16	31	175	3.9
K75	8	5	9	8	9	6	9	9	9	9	6	6	3	3	79	5	37	0.9	30	21	38	105	4.2
K76	8	5	9	8	9	6	9	9	9	9	6	9	3	3	71	3	42	0.8	29	19	42	120	4.8
K77	8	6	6	8	8	6	6	8	9	9	6	9	3	3	61	3	41	0.9	24	20	33	130	3.7
K78	9	6	6	9	9	6	6	9	9	9	6	9	9	9	63	3	28	0.8	26	17	28	80	3.4
K79	9	6	8	9	9	6	7	9	9	9	6	9	3	3	78	3	46	1.0	30	21	37	185	6.6
K80	9	6	6	9	9	6	7	9	9	9	6	9	3	3	72	5	43	0.8	35	17	29	165	3.4
K81	9	6	6	9	9	6	6	9	9	9	7	9	3	3	82	5	44	1.2	43	25	48	185	3.6
K82	9	6	8	9	9	6	8	9	9	9	7	8	3	3	86	3	46	1.0	34	19	44	180	3.5
G93	6	8	8	6	6	8	8	6	9	9	7	3	5	5	27	3	68	1.1	37	24	27	320	22.8
K83	9	8	7	9	9	8	8	9	9	9	7	9	3	3	84	5	55	1.0	33	18	37	197	3.4
K84	9	6	8	9	9	6	8	9	9	9	7	9	3	3	82	5	52	1.0	41	18	27	215	3.2
K85	9	5	8	9	9	6	8	9	9	9	6	9	3	3	76	3	43	0.9	37	20	24	220	4.2
K86	9	6	8	9	9	7	7	9	9	9	7	9	3	3	61	3	34	0.7	28	22	39	180	3.4
K87	9	6	8	9	9	7	7	9	9	9	7	9	3	3	55	5	40	0.9	34	25	39	235	3.4
K88	9	6	8	9	9	7	7	9	9	9	7	9	3	3	56	5	52	1.1	42	27	29	135	4.2
K89	9	6	8	9	9	7	7	9	9	9	7	9	3	3	52	3	62	0.9	32	22	28	165	4.0
K90	9	7	8	9	9	7	7	9	9	9	7	9	9	9	57	3	56	1.0	38	18	34	405	5.0
K91	9	7	8	9	9	7	7	9	9	9	7	9	9	9	54	5	52	0.9	36	18	41	150	3.7
K92	8	7	7	8	8	8	7	8	9	9	8	9	5	5	53	3	54	1.1	41	23	44	290	7.9
K93	7	8	9	7	7	8	7	7	9	9	9	9	5	5	70	3	42	1.0	33	29	18	275	5.7
G105	6	6	6	6	6	7	6	6	5	5	6	3	9	9	10	3	12	0.7	11	11	16	35	3.5
G106	7	7	6	7	7	7	6	7	7	6	6	3	9	9	26	3	25	0.8	28	13	18	35	3.4
G107	5	7	6	5	6	7	6	6	7	6	7	4	9	9	18	3	15	0.8	15	13	16	20	3.1
G108	6	7	6	6	7	7	6	7	7	7	7	1	9	9	20	3	27	0.7	28	22	21	20	3.5
G109	6	7	6	6	7	7	6	7	7	7	7	3	9	9	30	3	20	0.7	14	14	19	45	3.3
G110	6	8	9	6	6	8	8	6	8	8	7	7	5	5	26	3	25	0.7	20	10	26	70	3.3
K94	7	8	6	7	7	8	8	7	7	7	6	6	9	9	39	3	36	0.8	22	26	36	50	3.4
G112	6	7	6	6	7	8	8	7	7	7	6	6	5	5	25	3	25	0.8	20	21	19	25	3.3
G113	6	7	6	6	7	8	8	7	7	7	6	6	5	5	32	3	20	0.9	17	18	27	35	3.4
G114	6	8	6	6	7	8	8	7	7	7	6	6	9	9	24	3	22	0.6	14	12	16	20	3.2
G115	5	7	6	5	5	8	5	5	5	5	5	5	9	9	20	3	23	0.8	18	22	17	34	3.4
G116	6	5	6	6	6	6	8	6	7	7	4	4	9	9	29	3	20	0.5	11	12	13	70	3.3
K95	7	7	6	7	8	8	8	8	7	7	7	8	5	5	51	3	25	0.8	25	26	27	100	4.2
G118	5	7	6	5	5	8	6	5	7	6	5	2	9	9	20	3	10	0.7	12	13	15	5	3.4
G119	5	7	6	5	5	8	5	5	5	5	2	1	9	9	19	3	15	0.7	16	14	17	10	3.5
G120	5	5	5	5	5	7	8	5	5	5	4	1	9	9	16	3	12	0.7	11	10	11	5	3.3
G121	6	7	7	6	6	8	8	6	6	6	5	6	9	9	25	3	19	0.9	14	14	17	121	3.4
G122	6	7	7	6	6	8	8	6	7	7	5	6	9	9	24	3	20	0.7	14	13	16	20	3.4
G123	6	7	6	6	6	8	8	6	6	6	5	1	9	9	19	3	10	0.6	17	17	16	10	3.9
K96	7	7	8	7	7	7	8	7	7	7	5	5	9	9	14	3	18	0.5	26	22	26	15	3.3
G125	6	7	5	6	6	7	6	6	5	5	5	6	9	9	11	3	12	0.7	13	10	12	15	3.8
G126	6	7	6	6	6	7	6	6	5	5	4	4	9	9	25	3	13	0.4	11	10	12	25	3.3
G127	7	7	6	7	7	7	6	7	5	5	4	1	9	9	22	3	12	0.4	11	10	12	5	3.2

G128	6	6	5	6	6	7	5	6	7	7	5	2	9	9	20	3	10	0.8	17	15	22	5	3.8
G129	5	6	6	5	5	7	5	5	7	7	5	5	9	9	19	3	13	0.9	22	26	36	16	3.3
G130	7	6	5	7	7	7	5	7	7	7	6	8	5	5	33	3	27	0.7	18	18	22	40	3.8
G131	6	7	5	6	7	7	5	7	7	7	6	5	5	5	25	3	27	0.2	13	17	20	30	3.2
G132	6	6	6	6	6	6	6	6	5	5	6	1	9	9	21	3	14	0.4	11	12	12	10	4.7
G133	6	6	6	6	6	6	6	6	5	5	6	1	9	9	19	3	5	0.5	5.5	8	10	5	4.6
G134	6	7	5	6	6	7	6	6	5	5	6	1	9	9	15	3	10	0.6	9.5	10	11	10	3.1
G135	6	7	6	6	6	7	6	6	7	7	6	5	5	5	29	3	23	0.9	19	23	22	25	4.2
G136	6	7	6	6	6	8	6	6	7	7	6	2	9	9	24	3	21	0.6	24	25	26	20	4.1
G137	6	6	7	6	6	6	7	6	5	5	5	5	5	5	19	3	14	0.8	17	15	22	15	3.3
G138	5	7	7	5	6	7	7	6	5	5	5	1	9	9	17	3	12	0.6	16	11	14	10	4.2
G139	5	7	6	5	5	7	5	5	5	5	5	5	9	9	7	3	8	0.3	10	10	12	12	4.4
G140	5	5	6	5	6	7	5	6	5	5	5	5	9	9	14	3	15	0.4	11	10	12	10	3.2
G141	5	7	5	5	6	7	7	6	5	5	6	1	9	9	19	3	12	0.5	9	9	10	15	3.1
G142	6	7	7	6	7	7	7	7	7	7	6	8	5	5	29	3	30	0.9	24	22	25	40	4.2
G143	6	5	6	6	6	7	5	6	5	4	7	1	9	9	17	3	5	0.8	34	23	22	15	4.1
G144	5	7	5	5	6	7	6	6	5	5	7	1	9	9	19	3	12	0.7	19	16	19	15	3.3
G145	5	7	6	5	6	7	6	6	5	5	7	1	9	9	16	3	10	0.5	12	11	11	5	3.8
G146	5	7	6	5	5	7	5	5	6	6	5	5	9	9	17	3	10	0.5	12	10	12	13	3.2
G147	5	7	6	5	5	7	5	5	6	6	5	5	9	9	13	3	10	0.5	12	10	12	11	4.7
G148	5	7	6	5	6	7	6	6	6	6	7	1	9	9	21	3	20	0.6	16	15	16	10	3.3
G149	5	7	6	5	5	7	6	5	6	6	8	1	9	9	18	3	21	0.9	23	25	31	15	3.3
G150	6	6	6	6	6	6	6	6	6	6	5	5	9	9	27	3	18	0.5	26	17	23	86	7.3
G151	6	7	7	6	6	7	8	6	7	7	8	2	9	9	22	3	20	0.9	20	11	25	20	3.4
G152	6	7	6	6	6	8	6	6	7	7	7	1	9	9	23	3	14	0.7	16	12	14	15	3.4
K97	7	8	6	7	7	8	8	7	7	7	6	6	9	9	32	3	30	0.8	23	25	35	30	3.0
K98	7	8	9	7	7	8	8	7	8	8	8	7	5	5	36	3	30	0.7	21	18	25	105	3.4
G155	6	6	6	6	6	6	6	6	5	5	5	5	9	9	26	3	13	0.6	11	10	11	10	3.4
G156	6	6	5	6	6	6	6	6	5	5	5	5	9	9	24	3	5	0.8	34	28	16	10	3.6
G157	6	7	7	6	7	8	7	7	7	7	5	3	9	9	28	1	22	0.4	22	21	21	20	3.2
G158	6	7	7	6	6	8	7	6	8	7	7	2	9	9	25	3	20	0.8	25	20	19	150	3.4
K99	7	8	8	7	7	8	9	7	8	8	7	7	5	5	35	3	25	0.9	25	21	21	100	3.3
G160	6	7	7	6	6	7	7	6	6	8	8	1	9	9	86	3	40	1.0	33	21	29	45	3.2
K100	7	8	7	7	8	8	8	8	7	8	6	8	9	9	34	3	25	0.7	35	25	34	75	3.4
G162	6	8	7	6	7	7	7	7	7	8	6	8	9	9	30	3	27	0.8	30	23	31	205	5.2
G163	6	6	5	6	7	7	5	7	6	6	5	1	9	9	24	3	13	0.9	20	11	17	15	6.2
G164	6	6	5	6	7	7	7	7	6	6	7	4	9	9	30	3	24	0.7	19	26	20	25	3.4
K101	7	7	9	7	7	7	8	7	8	8	7	5	9	9	28	3	20	0.7	20	26	24	50	5.6
K102	7	8	8	7	7	9	8	7	8	8	5	7	5	5	37	3	25	0.6	23	15	22	14	4.3
G167	7	6	6	7	7	7	6	7	8	8	6	6	5	5	37	3	30	0.8	23	13	24	30	3.4
G168	7	7	7	7	7	7	6	7	8	8	6	8	5	5	29	3	35	0.9	27	24	27	85	3.6
G169	5	6	6	5	6	7	7	6	7	7	6	5	9	9	24	3	25	0.7	22	18	24	35	3.4
G170	5	6	6	5	6	6	7	6	7	7	6	5	5	5	22	3	20	0.6	19	10	18	35	6.3
K103	8	8	9	8	8	9	9	8	9	9	7	7	5	5	63	3	40	1.0	37	22	32	65	19.5
G172	7	7	7	7	7	7	9	7	6	6	6	2	9	9	19	3	19	0.6	14	13	15	24	4.6
G173	6	6	6	6	6	6	6	6	6	6	5	5	9	9	16	3	10	0.6	11	16	19	17	3.3

G174	5	5	6	5	5	5	6	5	5	5	5	5	9	9	20	3	18	0.5	26	24	28	90	3.6
G175	5	5	6	5	6	5	6	6	6	5	5	5	9	9	13	3	27	0.6	23	25	34	98	3.3
G176	5	5	6	5	6	6	6	6	6	5	5	5	9	9	11	3	20	0.7	20	25	20	45	3.3
K104	7	7	8	7	7	7	8	7	7	7	5	5	5	5	39	3	22	0.7	20	26	21	40	3.6
G178	6	7	5	6	6	7	6	6	5	5	5	6	5	5	19	3	16	0.8	22	25	35	30	3.2
G179	6	7	8	6	7	7	8	7	7	7	5	5	9	9	26	3	27	0.6	22	18	20	30	3.3
G180	6	6	6	6	7	7	8	7	7	7	5	3	5	5	29	3	20	1.2	17	12	15	40	3.2
G181	5	5	5	5	6	6	6	6	7	7	5	5	9	9	26	3	20	1.2	17	10	14	40	3.4
G182	6	7	7	6	7	7	7	7	7	7	5	3	5	5	30	3	27	0.4	26	11	14	120	1.1
G183	7	7	7	7	7	7	6	7	7	7	5	5	9	9	47	3	27	0.4	26	20	19	25	3.4
K105	7	7	7	7	7	8	7	7	8	8	6	8	5	5	35	3	26	0.9	23	23	26	65	3.3
G185	6	6	5	6	7	7	5	7	6	6	5	5	9	9	28	3	5	0.2	23	24	28	15	3.3
G186	6	7	7	6	6	7	8	6	6	6	5	5	9	9	18	3	5	0.2	23	25	34	15	3.3
K106	7	7	8	7	7	8	8	7	8	7	4	7	9	9	37	3	29	0.3	20	10	10	45	3.2
G188	6	6	6	6	6	6	6	6	6	6	5	5	9	9	28	3	18	0.5	26	17	23	86	3.3
G189	6	6	6	6	6	6	6	6	6	6	5	5	9	9	28	3	18	0.5	26	18	24	87	3.3
K107	7	7	9	7	7	7	9	7	8	7	5	5	5	5	36	3	24	0.7	19	18	25	60	3.3
K108	7	7	9	7	7	7	9	7	8	7	5	7	5	5	31	3	25	0.6	25	18	24	85	3.3
K109	8	8	9	8	7	8	9	7	8	8	6	1	5	5	35	3	30	0.6	21	15	14	45	4.1
G193	8	8	9	8	7	8	9	7	9	8	6	9	5	5	43	3	30	0.7	28	15	14	95	3.2
K110	8	8	9	8	7	8	9	7	9	8	6	7	9	9	46	3	30	0.6	30	17	14	85	3.3
K111	8	8	9	8	7	8	9	7	9	8	7	2	5	5	41	3	31	0.7	23	15	14	65	3.4
K112	6	8	8	6	6	8	8	6	9	8	8	7	5	5	38	3	33	0.9	29	25	31	15	3.4
G197	6	6	6	6	6	6	6	6	6	6	5	5	9	9	27	3	18	0.5	26	18	24	87	6.2
G198	6	7	7	6	7	8	7	7	8	8	6	6	9	9	53	3	49	0.9	48	26	35	85	6.5
G199	5	8	7	5	6	8	7	6	9	9	7	9	9	9	68	3	40	0.7	25	31	20	55	4.5
K113	8	8	9	8	7	8	9	7	8	8	6	1	5	5	24	3	30	0.6	21	15	14	45	3.3
K114	8	8	9	8	7	8	9	7	9	8	6	9	5	5	43	3	30	0.7	28	15	14	95	3.3
K115	8	8	9	8	7	8	9	7	9	8	6	7	9	9	45	3	30	0.6	30	17	14	85	4.1
K116	8	8	9	8	7	8	9	7	9	8	7	2	5	5	41	3	31	0.7	23	15	14	65	3.5
G204	6	6	6	6	6	6	6	6	6	6	5	5	9	9	28	3	18	0.5	26	17	23	86	3.4
G205	6	6	6	6	6	6	6	6	6	6	5	5	9	9	25	3	18	0.5	26	18	24	87	3.8
G206	6	8	7	6	7	7	7	7	8	8	6	6	9	9	48	3	40	0.7	39	18	32	270	5.7
G207	8	5	7	8	9	6	7	9	9	9	6	9	3	3	41	3	70	0.9	59	23	20	360	7.8
K117	8	8	9	8	7	8	9	7	8	8	6	1	5	5	34	1	30	0.6	21	15	14	45	3.4
K118	8	8	9	8	7	8	9	7	9	8	6	9	5	5	43	1	30	0.7	28	15	14	95	3.5
K119	8	8	9	8	7	8	9	7	9	8	6	7	9	9	45	1	30	0.6	30	17	14	85	3.4
K120	8	8	9	8	7	8	9	7	9	8	7	2	5	5	41	1	31	0.7	23	15	14	65	3.8
G212	6	6	6	6	6	6	6	6	6	6	5	5	9	9	26	3	18	0.5	26	17	23	86	3.2
G213	6	9	9	6	6	7	7	6	7	7	9	9	5	5	22	3	49	1	37	28	31	315	47.7
G214	6	6	7	6	7	7	8	7	7	8	7	9	5	5	49	3	35	1.0	37	19	25	95	9.6
G215	7	8	8	7	8	8	9	8	7	7	7	8	5	5	53	3	35	0.9	32	21	34	135	9.9
G216	6	7	8	6	6	7	8	6	8	8	7	2	9	9	50	3	40	1.0	30	19	21	40	10.2
G217	6	9	9	6	6	7	8	6	7	7	9	9	5	5	57	3	50	1.0	36	18	32	200	20.9
G218	8	8	9	8	7	8	9	7	9	8	6	7	9	9	41	3	30	0.6	30	17	14	85	3.4
G219	6	8	8	6	6	7	7	6	8	9	9	7	9	9	56	3	45	0.8	33	18	31	160	14.6

G220	6	6	8	6	6	8	8	6	6	6	8	8	9	9	30	3	27	0.8	23	21	25	105	8.6
G221	6	9	8	6	6	9	7	6	8	9	9	1	9	9	40	3	60	1.0	41	28	32	110	9.5
G222	6	7	8	6	6	7	8	6	6	6	7	7	5	5	22	3	37	0.8	30	26	28	25	14.9
G223	6	7	6	6	6	8	6	6	7	8	9	1	9	9	61	3	40	0.9	32	25	25	85	6.5

Table S4. USDA accession numbers and countries of origin of the 120 tall fescue genotypes selected for SSR analysis.

No	Accession	Country of origin	Initial seedlings (n)	Surviving genotypes (n)	Survival (%)	Genotype code
**1**	PI634232	Bulgaria	20	7	35	K1, K2, K3, K4, K5
**2**	PI199249	Greece	20	1	5	K6
**3**	PI249738	Greece	20	20	100	K7, K8, K9, K10, K11, K12, K13, K14, K15, K16, K17, K18, K19
**4**	PI504538	Greece	20	1	5	K20
**5**	PI289004	Hungary	20	18	90	K21, K22, K23, K24, K25, K26, K27, K28, K29, K30, K31, K32, K33, K34, K35, K36, K37, K38
**6**	PI598492	Hungary	20	6	30	K39, K40, K41, K42, K43, K44
**7**	PI598494	Hungary	20	7	35	K45, K46, K47, K48, K49, K50, K51
**8**	PI598495	Hungary	20	2	10	K52, K53
**9**	PI598496	Hungary	20	1	5	K54
**10**	PI598497	Hungary	20	12	60	K55, K56, K57, K58, K59, K60, K61, K62, K63, K64, K65, K66
**11**	PI311044	Romania	20	2	10	K67, K68
**12**	PI595072	Romania	20	2	10	K69, K70
**13**	PI598493	Romania	20	1	5	K71
**14**	PI659825	Romania	20	20	100	K72, K73, K74, K75, K76, K77, K78, K79, K80, K81, K82, K83, K84, K85, K86, K87, K88, K89, K90, K91
**15**	W610027	Romania	20	0	0	–
**16**	PI184041	Serbia	20	1	5	K92
**17**	PI172423	Türkiye	20	16	80	K93
**18**	PI174209	Türkiye	20	14	70	K94, K95
**19**	PI174210	Türkiye	20	20	100	–
**20**	PI204446	Türkiye	20	20	100	K96, K97, K98, K99, K100, K101, K102, K103
**21**	PI204447	Türkiye	20	20	100	K107, K108, K109, K110, K111, K112, K113, K114, K115, K116
**22**	PI206270	Türkiye	20	16	80	K104, K105, K106
**23**	PI383647	Türkiye	20	0	0	–
**24**	PI577088	Montenegro	20	16	80	K117, K118, K119, K120
		Total/Mean	480	223	46.4	

Table S5. Ranking of 223 tall fescue genotypes based on the multi-trait weighted selection index.

No	Genotype	Index (0-100)	No	Genotype	Index (0-100)	No	Genotype	Index (0-100)	No	Genotype	Index (0-100)	No	Genotype	Index (0-100)	No	Genotype	Index (0-100)	No	Genotype	Index (0-100)
1	K69	85.2	33	K14	73.2	65	K61	70	97	K29	64.8	129	G198	58.1	161	G108	49.7	193	G188	40.4
2	K70	83.3	34	K75	73.2	66	K85	70	98	K92	64.8	130	G114	57.9	162	G109	49.5	194	G189	40.4
3	K103	81.7	35	K88	73.1	67	K78	69.9	99	K51	64.7	131	G172	57.9	163	G127	49.5	195	G204	40.4
4	K65	79.3	36	K45	73	68	K50	69.8	100	K108	64.5	132	G183	56.8	164	G220	49.4	196	G205	40.4
5	K64	78.9	37	K118	72.8	69	K60	69.7	101	K4	64.5	133	G199	56.6	165	G136	49.3	197	G212	40.3
6	K56	77.7	38	K76	72.8	70	K102	69.1	102	K52	64.5	134	G168	56.5	166	G160	48.3	198	G148	40.1
7	K35	77.6	39	K86	72.5	71	K5	68.3	103	K48	64.4	135	G157	56.4	167	G130	47.9	199	G178	39.5
8	K68	77.4	40	K111	72.4	72	K25	68.2	104	K8	64.4	136	G93	56.2	168	G152	47.9	200	G134	39.3
9	K83	77.3	41	K116	72.4	73	K53	68.2	105	K31	64.1	137	G216	56.1	169	G223	47.1	201	G105	39.1
10	K7	76.9	42	K18	72.4	74	K80	68.2	106	K55	63.9	138	G25	56	170	G131	46.3	202	G155	38.8
11	K91	76.6	43	K19	72.4	75	K41	67.7	107	K30	63.8	139	G122	55	171	G135	46.1	203	G129	38.2
12	K43	76.4	44	K20	72.4	76	K6	67.7	108	K1	62.7	140	G112	54.9	172	G14	46	204	G132	38
13	K9	76.4	45	K46	72.4	77	K100	67.4	109	K17	62.6	141	G113	54.9	173	G164	45.9	205	G133	38
14	K42	76	46	K38	72.3	78	K58	67.3	110	K93	62.5	142	G219	54.9	174	G116	45.8	206	G141	37.7
15	K44	75.4	47	K34	72.2	79	K32	67	111	K96	62.2	143	G217	54.3	175	G218	45.5	207	G156	37.4
16	K71	75	48	K82	72.1	80	K37	67	112	K97	61.6	144	G162	54.1	176	G1	45.3	208	G145	37.3
17	K115	74.8	49	K120	72	81	K77	66.8	113	K49	61.4	145	G206	54	177	G185	44.6	209	G119	36.7
18	K10	74.7	50	K72	72	82	K26	66.4	114	K94	61.3	146	G167	53.9	178	G73	44.3	210	G181	36.7
19	K11	74.7	51	K22	71.9	83	K57	66.4	115	K47	61.2	147	G142	53	179	G169	43.8	211	G147	36.6
20	K110	74.7	52	K62	71.9	84	K3	66.3	116	K105	60.6	148	G213	53	180	G7	43.1	212	G146	36.4
21	K59	74.7	53	K89	71.9	85	K39	66.1	117	K23	60.6	149	G214	53	181	G163	43	213	G149	36.1
22	K67	74.7	54	K109	71.8	86	K16	65.9	118	K104	60.5	150	G106	52	182	G128	42.8	214	G144	35.5
23	K54	74.6	55	K113	71.6	87	K99	65.8	119	K95	60.5	151	G34	51.9	183	G126	42.6	215	G176	35
24	K12	74.6	56	K36	71.6	88	K33	65.7	120	K112	60.2	152	G186	51.5	184	G138	41.9	216	G115	34.9
25	K15	74.6	57	K87	71.5	89	K81	65.7	121	G215	60	153	G75	51.3	185	G173	41.9	217	G143	34.3
26	K40	74.4	58	K84	71.3	90	K106	65.4	122	G193	59.9	154	G180	50.8	186	G107	41.7	218	G120	33.9
27	K66	74.3	59	K117	71.2	91	K2	65.4	123	G23	59.8	155	G182	50.8	187	G118	41.5	219	G139	33.8
28	K119	74.2	60	K79	71.2	92	K101	65.2	124	G207	59.6	156	G222	50.8	188	G137	40.8	220	G140	33.8
29	K74	73.5	61	K90	70.5	93	K98	65.1	125	G221	59.6	157	G151	50.6	189	G150	40.8	221	G175	32.3
30	K13	73.3	62	K24	70.4	94	K27	65	126	G110	59.3	158	G158	50.4	190	G170	40.8	222	G18	31.2
31	K21	73.2	63	K73	70.4	95	K107	64.9	127	G81	58.8	159	G123	50.3	191	G197	40.7	223	G174	27.3
32	K114	73.2	64	K63	70.1	96	K28	64.9	128	G179	58.2	160	G121	49.8	192	G125	40.4			

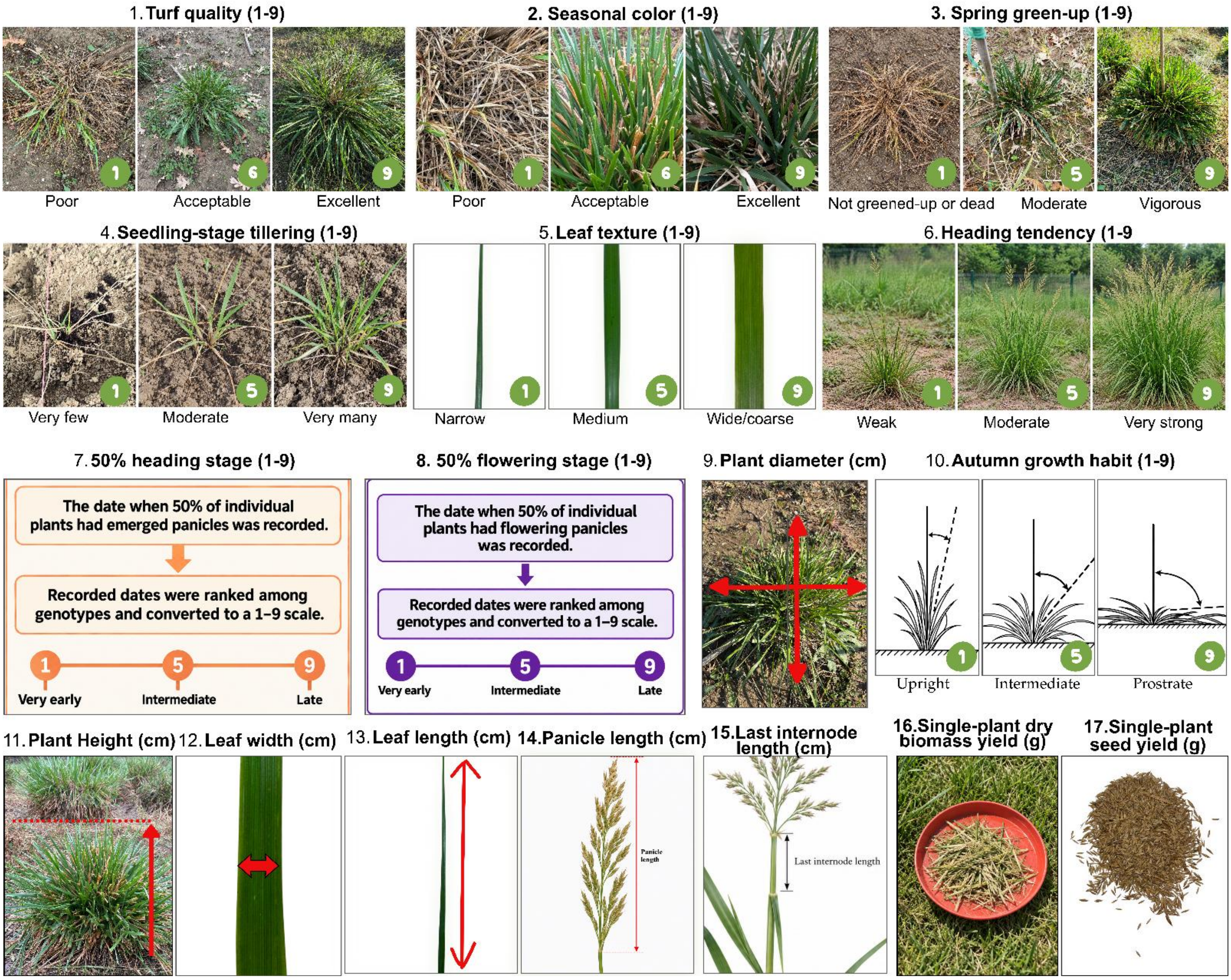

Figure S1. Representative images and schematic illustrations showing the evaluation methods for the 17 phenotypic and turf-related traits assessed in tall fescue.
